# Bioactive Natural Products: Facts, Applications, and Challenges

**DOI:** 10.1155/2015/684109

**Published:** 2015-04-15

**Authors:** Yiannis Kourkoutas, Kimon A. G. Karatzas, Vasilis P. Valdramidis, Nikos Chorianopoulos

**Affiliations:** ^1^Applied Microbiology and Molecular Biotechnology Research Group, Department of Molecular Biology and Genetics, Democritus University of Thrace, 68100 Alexandroupolis, Greece; ^2^Department of Food and Nutritional Sciences, University of Reading, Reading RG6 6AD, UK; ^3^Department of Food Studies and Environmental Health, Faculty of Health Sciences, University of Malta, Msida MSD 2080, Malta; ^4^Institute of Technology of Agricultural Products, Greek Agricultural Organization Demeter, 15310 Athens, Greece

Today, there are a strong debate and interest regarding the safety aspects of chemical preservatives added widely in many food products to prevent mainly growth of spoilage and pathogenic microbes. Synthetic compounds are considered responsible for carcinogenic and teratogenic attributes and residual toxicity. To avoid the aforementioned problems, consumers and authorities have increased pressure on food manufacturers to substitute the harmful artificial additives with alternative, more effective, nontoxic, and natural substances. In this context, the use of natural compounds with antimicrobial action presents an intriguing case. Natural antioxidants also demonstrate a wide range of biological and pharmacological activities and are considered to have beneficial effects in nutrition and health [1, 2]. Natural products are currently used in several product preparations mainly as flavouring agents, fragrances, and functional additives by the cosmetic and pharmaceutical industries [3], while their individual components are also used as flavourings [4]. These natural substances have been suggested for use in foodstuffs [5], as they are known to display significant antimicrobial properties [6–8].

In order to extend our knowledge on the effectiveness of natural bioactive products and explore their application as antimicrobial systems and in functional foods production, research must be focused on the following issues: the elucidation of the molecular cell mechanisms through which microorganisms respond against natural bioactive products; the definition of matrix effects on the antimicrobial efficiency of a natural bioactive product in combination with other hurdles; the use of emerging technologies in combination with natural products, which may act synergistically for microbial growth prevention; the determination of other biological activities of natural products, for example, those relative to antioxidant and anticancer potential, and the identification of possible mechanism(s) of action; the understanding of consumer attitudes and quality perception.

Additionally, more emphasis should be given on prevalence assays of pathogenic microorganisms in connection with the use of natural antimicrobials during various production stages in industry. The inclusion of several factors, such as matrix and physiological stage of microorganisms, into mathematical models describing microbial growth and death, would represent a significant advancement in quantitative studies when compared with the empirical, descriptive models of microbial growth of limited predictive capability, currently used by the industries [9–11].

The main objective of this special issue is to provide a number of documents focused on the facts, applications, and challenges of bioactive natural products and present the methodologies in use for their effectiveness evaluation. Moreover, the challenges that industry faces with respect to the use of bioactive natural products as antimicrobial agents in terms of safety and microbial growth prevention are discussed. A better understanding of the proposed mechanisms of action for some natural compounds and relevant key molecular factors in bacterial biofilm formation and their regulation, such as the chemical signalization machinery involved in bacteria-environment interaction, are also referred to. Furthermore, the application of high hydrostatic pressure treatment as a reliable nonthermal pasteurization method to extend the microbiological shelf life of various foodstuffs is thoroughly discussed. Finally, the potential of various plant-derived compounds to control pathogenic bacteria and especially the diverse effects exerted by plant compounds on virulence factors that are critical for pathogenicity is highlighted and assessed.

*Yiannis Kourkoutas* *Kimon A. G. Karatzas* *Vasilis P. Valdramidis* *Nikos Chorianopoulos*
